# Concurrent Transmission Based on Distributed Scheduling for Underwater Acoustic Networks

**DOI:** 10.3390/s19081871

**Published:** 2019-04-19

**Authors:** Jun Zhang, Hanhua Lai, Yan Xiong

**Affiliations:** 1 School of Electronic and Information Engineering, South China University of Technology, Guangzhou 510641, China; 18825157036@163.com; 2 Department of Computer Science, Guangdong University of Education, Guangzhou 510303, China; xiongy@gdei.edu.cn

**Keywords:** underwater acoustic networks, handshaking, concurrent transmission, distributed scheduling

## Abstract

Handshaking is a common technique used to avoid collisions in terrestrial and underwater content-based networks. However, due to the long propagation delay of the underwater acoustic channel, the conventional handshaking mechanism, which only allows one successful handshake and one pair of nodes to communicate per transmission cycle, becomes less effective in underwater acoustic networks. This paper proposes a new distributed scheduling method for underwater acoustic networks that supports multiple handshakes and concurrent transmissions in one transmission cycle for one-hop clusters. A deterministic scheduling algorithm was developed to optimize the sending sequence and time of the source nodes jointly so that the total data transmission time is shortened while avoiding collisions among multiple concurrent transmissions. The deterministic scheduling algorithm can also reduce the scheduling overhead and enables the synchronization of the data concurrent transmissions in a distributed manner via the standard two-way handshaking. Simulation results show that the proposed method outperforms several conventional underwater medium access control protocols in normalized throughput, packet delivery rate, average end-to-end delay, and average energy consumption.

## 1. Introduction

As the need for monitoring the marine environment for scientific exploration, commercial exploitation and coastline protection continues to grow, underwater acoustic communication and networking are receiving considerable attention [1,2,3]. Underwater acoustic channels, which are characterized by limited bandwidth, high and variable propagation delay, time-varying multi-path and fading, resulting in high bit error rates and temporary losses of connectivity, creates challenges for underwater acoustic networking [3]. The medium access control (MAC) protocol, which controls the nodes in a network to share a common broadcast channel, is one of the key techniques for underwater acoustic networking.

Many MAC protocols for underwater acoustic networks have been proposed [1,2,3]. They can be broadly classified into three categories: contention-free, contention-based and hybrid protocols [2]. In the contention-free protocols, a node does not need to compete with other nodes when accessing the medium. Collisions are avoided by assigning different frequency bands, time slots, or spreading codes to different users. Typical contention-free schemes include frequency division multiple access (FDMA), time division multiple access (TDMA), and code division multiple access (CDMA). In the contention-based protocols, the nodes compete for the opportunity to access the medium. Because contention causes collisions which significantly reduce the efficiency of the communication, minimizing the collisions is the key task for contention-based protocols. Besides, the required overheads for the contentions should also be considered and minimized. Random access and handshaking are two major schemes used in contention-based protocols. Usually, contention-free protocols are suitable for continuous transmission, whereas contention-based protocols are more efficient for burst data transmission. To take the advantage of both techniques, the hybrid MAC protocols integrate different medium access techniques so that better performance can be achieved in underwater environments.

Handshaking is a technique widely used to avoid collision in contention-based terrestrial networks and was standardized in IEEE 802.11i. However, the conventional handshaking mechanism, which only allows one successful handshake and one pair of nodes to communicate per transmission cycle, becomes less effective in underwater environments due to the long propagation delay of underwater acoustic channels. Exchanging request-to-send (RTS) and clear-to-send (CTS) packets in the underwater environment requires much more time than in terrestrial radio channels, so the transmission efficiency decreases significantly. Many modified handshake-based protocols have been proposed to address this problem. One of the most effective methods is to exploit the opportunity of the temporal and/or spatial reuse of the acoustic channel that is created by the long propagation latency to allow multiple concurrent packets to propagate in the same contention domain [4]. In the previous research, several handshake protocols with temporal and/or spatial reuse techniques have been developed in sender- or receiver-initiated modes for centralized or distributed underwater acoustic networks [4,5,6,7,8,9,10]. However, little attention has been paid to using this technique to implement multiple handshakes and multiple concurrent transmissions in one transmission cycle for distributed underwater acoustic networks. Besides, the sending sequence of the source node affects the transmission efficiency due to the different propagation delays between the source and destination nodes, which has not been mentioned in previous studies.

In this paper, we propose a new distributed scheduling method for underwater acoustic networks that supports multiple handshakes and concurrent transmissions in one transmission cycle for one-hop clusters using the temporal reuse technique. The contributions of our work include:

- A new synchronization method for distributed scheduling was developed, whose basic idea involves scheduling using a deterministic algorithm. Because deterministic algorithms can generate the same results independently when given identical initial conditions, the synchronization for distributed scheduling can be achieved via a two-way handshake, which is the simplest form of the handshaking protocol and has the least overhead.
- The influence of the sending sequence of the source nodes on the concurrent transmission efficiency was discussed. A scheduling method based on dynamic programming for the joint optimization of the node’s sending sequence and time is proposed. By considering the sending sequence of the source nodes, the transmission efficiency can be further improved.
- Based on the above two methods, we propose a new distributed scheduling MAC protocol for underwater acoustic networks that supports multiple handshakes and concurrent transmissions in one transmission cycle for one-hop clusters. By supporting more concurrent transmission in a transmission cycle, the proposed method outperforms several conventional underwater MAC protocols in the simulations.

The rest of this paper is organized as follows: in Section 2, the works related to underwater MAC protocols are reviewed. In Section 3, the new distributed scheduling method for underwater acoustic networks is proposed. Section 4 and Section 5 present and discuss the simulation results, respectively. Finally, Section 6 concludes the paper.

## 2. Related Work

Many MAC protocols have been proposed for underwater acoustic networks [1,2,3]. In Chen et al. [2], these protocols were divided into three categories: contention-free, contention-based, and hybrid protocols. Contention-free MAC protocols divide the spectral resources into discrete channels and separately assign them to nodes to avoid contentions. FDMA assigns different frequency bands to individual users. However, the limited bandwidth of underwater acoustic channels greatly restricts the scale of network. To overcome this problem, orthogonal frequency division multiple access (OFDMA) techniques [11,12] for underwater acoustic networks were proposed to improve the use of the limited bandwidth. OFDMA allows multiple user symbols to transmit simultaneously using different subcarriers with mutually orthogonal overlapping frequency bands so that more users can be supported. CDMA [13,14] is a physical and MAC layer technique that allows multiple users to communicate simultaneously in the same frequency band by spreading the user messages with pseudo-noise (PN) codes. The CDMA-based MAC protocols in underwater acoustic networks mainly focus on spreading code assignment, power control and energy efficiency. TDMA allows users to occupy the whole channel in non-overlapping time intervals. It is simple in implementation, so it is widely used in practical underwater acoustic networks. However, due to the large propagation delay in acoustic channels, the conventional TDMA protocol has lower channel use in underwater acoustic networks. Therefore, some modified TDMA protocols were proposed to improve the efficiency of the conventional TDMA through temporal and/or spatial reuse [15,16,17,18,19].

In the contention-based protocols, all nodes compete to assess a shared channel, which means that a node has the chance to exploit the full bandwidth of the channel when it wins in the contention, but will lose the chance for communication in the current transmission cycle when it fails in the contention. The contention-based protocols can be further divided into random access and handshaking protocols [2]. Random access protocols allow a node to transmit its data when needed. The ALOHA based protocols are typical random access protocols. In the basic version of ALOHA, a node sends data whenever it needs without any control. It is simple and easy to implement but many collisions occur when the traffic load is heavy. Some variances based on ALOHA have been proposed to improve its performance in underwater environments, such as Slotted ALOHA, propagation delay tolerant ALOHA, ALOHA with carrier sense, and ALOHA with advance notification [1,2,3]. Another type of widely used random access protocol is Carrier Sense Multiple Access (CSMA), in which all nodes have to sense the channel before sending data to reduce collisions. Improvements can be achieved by optimizing sensing duration, back-off window, scheduling, enabling interleaving, and energy-efficiency [1,2,3]. Without careful transmission controls, collisions become severe in the ALOHA- and CSMA-based protocols as the traffic loads increase.

To reduce collision, handshaking protocols use channel reservation to control the transmission of each node, in which a node has to capture the channel by handshaking before sending data. Floor Acquisition Multiple Access (FAMA) and Multiple Access Collision Avoidance (MACA) are two of the earliest handshaking protocols that use RTS and CTS for handshaking. New protocols were proposed based on FAMA and MACA by leveraging long propagation delays, improving handshaking efficiency, and improving handshaking success [2]. Among these improvements, using the long propagation delay to accommodate concurrent transmissions by scheduling or reservation is an effective method to improve overall performance. The MAC protocols using this technique include Reverse Opportunistic Packet Appending (ROPA) [5], Hybrid Sender- and Receiver-initiated (HSR) [6], Bidirectional-Concurrent MAC (BiC-MAC) [7], Delay-aware Opportunistic Transmission Scheduling (DOTS) [8], Receiver Initiated Packet Train (RIPT) [9], and Decoupled and Suppressed Handshaking (DSH-MAC) [10]. These protocols improve the transmission efficiency effectively; however, they still lack the ability to support multiple handshakes and concurrent transmissions for multiple pairs of senders and receivers in distributed networks.

In the hybrid MAC protocols, different medium access techniques are employed and can switch to each other on demands. Because the hybrid MAC protocols can take advantage of different medium access techniques, they usually produce better performance, so have become attractive research topics in underwater acoustic networks. Many hybrid MAC protocols have been proposed, which include the hybrid of contention-free protocols like the Hybrid Spatial Reuse Time-Division Multiple-Access (HSR-TDMA) [19], which combines the TDMA and CDMA techniques; the hybrid of contention-based protocols like the Underwater Practical MAC (UPMAC) [20], where ALOHA- and receiver-based protocols are used for low load mode and high load mode, respectively; and the hybrid of contention-free and contention-based protocols like Data-Collection-Oriented MAC (DCO-MAC) [21], where a contention-based MAC protocol is used in the sub-network with a light traffic load, whereas a reservation-based MAC protocol is used in the sub-network with a heavy traffic load.

## 3. Concurrent Transmission Based on Distributed Scheduling

### 3.1. System Description and Problems

Consider a one-hop cluster in a distributed underwater acoustic network, where all nodes in the cluster are static, randomly distributed and can reach each other directly using an omnidirectional and half-duplex underwater acoustic modem. The clocks in all nodes are synchronous. A delay table that contains the inter-node propagation delays in the cluster is stored by each node, which is obtained in the network initialization stage by measuring the round-trip time or exchanging information with its neighbors.

The basic idea of the proposed method is to allow multiple handshakes and concurrent transmissions in one transmission cycle so that the channel resource can be used more effectively. There are two major problems in designing such a MAC protocol:

- How to implement the scheduling algorithm in a distributed manner with the least overhead. Because there is no central node to broadcast the scheduling information, synchronizing the sending time of each source node to avoid collision is difficult.
- How to optimize the sending time of the source nodes to shorten the total data transmission time. The scheduling algorithm should ensure that a packet sent by an arbitrary source node will not overlap with other packets at its corresponding destination node (as a result, all destination nodes will receive their packets correctly), while ensuring the whole data transmission time is as short as possible. Note that overlaps of packets at their non-destination nodes are allowed.

### 3.2. Protocol Description

To solve the two problems addressed in Section 3.1, we propose a new distributed scheduling method that has the following features: (1) a deterministic scheduling algorithm is adopted to calculate the sending time table for each source node, through which a node can calculate its sending time table independently and obtain the same result when the input parameters of the scheduling algorithm are identical. This feature means that if the handshaking can provide all the input parameters for the scheduling algorithm, it is possible to synchronize the concurrent transmitted in distributed environments using a two-way handshake, which is the simplest form of the handshaking protocol and has the least overhead. (2) The sending sequence and time of the source nodes are optimized jointly using dynamic programming to ensure the packets sent by a source node do not conflict with other packets at its corresponding destination, while ensuring the data transmission time is as short as possible. This optimization algorithm is presented in Section 3.3.

**Figure 1:**
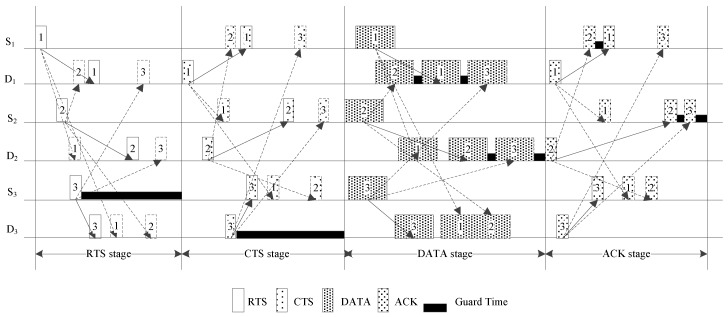
Timing diagram of the proposed protocol.

The proposed method follows the basic handshaking mechanism where the channel is reserved by the control packets of RTS and CTS. Time stamps are added to each control packet for synchronization. Because there is no central node to control the transmission, the source and destination nodes are synchronized through dedicated control packets, whose time stamps are unique in the transmission. A transmission cycle consists of four stages: the RTS stage, CTS stage, data stage, and acknowledgement (ACK) stage, as shown in Figure 1. In Figure 1, S_1_, S_2_, and S_3_ are the source nodes and D_1_, D_2_ and D_3_ are the corresponding destination nodes. The solid arrows represent the transmission to the recipient of a packet and the dashed arrows represent the transmissions to the other nodes that will also listen the packet.

#### 3.2.1. RTS Stage

The transmission starts from the RTS stage which is triggered by the first RTS packet sent by an arbitrary node in the cluster when the cluster is idle. Different from the conventional handshaking protocols, each node in the cluster can send out an RTS packet in this stage when it has data to transmit. It means that multiple RTS packets can be sent in the RTS stage. To reduce the collision of the RTS packets, the sending time of the following RTS packets are uniformly distributed in the time interval of $\left[T^{rts}-D_{i}^{rts},T_{0}\right]$, where *T^rts^* denote the time stamp of the last received RTS packet, $D_{i}^{rts}$ denotes the propagation delay of this RTS packet to the source node *s_i_*, and *T*_0_ is a constant used to control the length of the time interval, which is larger than the maximum propagation delay in the cluster. Each source node is allowed to send an RTS packet once in a transmission cycle to avoid a long RTS stage. When a node that sends an RTS does not receive the corresponding CTS, it waits for another transmission cycle instead of resending the RTS packet. The RTS stage ends after a fixed delay when the last RTS packet in the cluster is sent out.

#### 3.2.2. CTS Stage

When the RTS stage ends, all nodes in the cluster enter the CTS stage. In the CTS stage, the nodes that receive the RTS requests and are ready for receiving data will send out the CTS packets to their corresponding source nodes. The proposed method also allows multiple CTS packets to be sent in this stage. The CTS packets are sent out randomly within a time interval to reduce collisions. The CTS stage ends after a fixed delay when the last CTS packet is sent out.

#### 3.2.3. Data Stage

In the data stage, the source nodes that succeed in handshaking calculate their sending time and wait for transmission. Each source node independently executes a copy of the proposed scheduling algorithm. Because the proposed scheduling algorithm is deterministic and its input parameters are the node pairs that successfully shake hands in the current transmission cycle, all the source nodes can obtain the same sending time table for each source node through the proposed scheduling algorithm if they receive all the CTS packets correctly. When the sending time of a node arrives, it starts to transmit data to the corresponding destination node. Each node can independently calculate the ending time of the data stage using the proposed deterministic scheduling algorithm. Note that overlaps of data packets at their non-destination nodes are allowed in this stage, as shown in Figure 1, where the data packets from the source nodes S_1_ and S_2_ are allowed to overlap at the destination node D_3_.

#### 3.2.4. ACK Stage

The ACK/Negative ACKnowledgment (NACK) packets are sent using the same method as the data packets in the ACK stage. When all ACK/NACK packets are sent, the cluster turns idle and is ready for another new transmission cycle.

The fundamental difference between the proposed scheme and the basic RTS/CTS mechanism is that the former supports multiple successful handshakes and concurrent transmissions per transmission cycle, whereas the latter only allows one successful handshake and one pair of nodes to communicate. In the RTS and CTS stages, multiple RTS and CTS packets can be sent in the proposed scheme, which means that multiple successful handshakes are allowed. In the data and ACK stage, the data and ACK packets are transmitted concurrently at an optimized time. This batch transmission mode effectively makes use of the long propagation delay in underwater acoustic channels so that the total transmission time can be shortened when there is more than one node that has data to transmit. So, the proposed scheme is superior to the basic RTS/CTS mechanism in most cases.

### 3.3. Joint Optimization of Sending Sequence and Time

**Figure 2:**
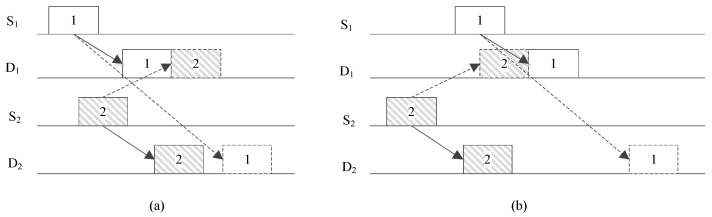
Concurrent transmissions with different sending sequences. (**a**) S_1_ sends first; (**b**) S_2_ sends first.

In previous studies, scheduling was usually implemented in a sender- and/or receiver-initiated manner for content-based networks, where a sender or receiver can receive multiple packets from different nodes. In these protocols, the effect of the sending sequence was insignificant. However, in the proposed protocol, multiple senders send packets to their corresponding receivers in a transmission cycle, which means the sending sequence is important to the transmission efficiency due to the unequal propagation delays among nodes. Figure 2 provides an example of two concurrent transmissions with different sending sequences. The total transmission time is much shorter when S_1_ sends the packet before S_2_. Therefore, in the proposed protocol, the sending sequence and times of the source nodes both must be optimized. In this section, we first consider the optimization of the sending time of the source node when the sending sequence is given. Then, a dynamic programming-based algorithm is proposed for the joint optimization of the sending sequence and time of the source nodes. To satisfy the synchronization requirement, no stochastic algorithm is used in the optimization.

#### 3.3.1. Sending Time Optimization

Assume that the sending sequence of the source nodes is given. Let *s_i_* and *s_j_* denote two arbitrary source nodes in a transmission cycle, *d_i_* and *d_j_* are their corresponding destinations. Assume that *s_i_* sends out its data packet before *s_j_* and the data packet from *s_i_* arrives at *d_i_* and *d_j_* earlier than the data packet from *s_j_*. Although this assumption might lead to sub-optimal solutions, it simplifies the discussion. To avoid collisions at *d_i_* and *d_j_*, the sending time of *s_i_* and *s_j_* should satisfied the following constraints:

- The data packets sent by *s_i_* and *s_j_* should not overlap at *d_i_*, which leads to:
  (1) $$T_{i}^{data}+L_{i}^{data}+D_{s_{i}d_{i}}\leq T_{j}^{data}+D_{s_{j}d_{i}},$$
  where $T_{i}^{data}$ and $T_{j}^{data}$ denote the sending time of the data packets from *s_i_* and *s_j_*, respectively. $L_{i}^{data}$ is the length of the data packet from *s_i_*; and $D_{s_{i}d_{i}}$ and $D_{s_{j}d_{j}}$ denote the propagation delays from *s_i_* to *d_i_* and *s_j_* to *d_i_*, respectively.
- The data packets sent by *s_i_* and *s_j_* should not overlap at *d_j_*, which leads to:
  (2) $$T_{i}^{data}+L_{i}^{data}+D_{s_{i}d_{j}}\leq T_{j}^{data}+D_{s_{j}d_{j}},$$
  where $D_{s_{i}d_{j}}$ and $D_{s_{j}d_{j}}$ denote the propagation delay from *s_i_* to *d_j_* and *s_j_* to *d_j_*, respectively.

Let *s*_1_ and *s_j_* denote the first and the *i*-th source nodes to send data packets in the current transmission cycle, respectively. At the beginning of the data stage, all source nodes reset their data stage clocks to the time stamp of the last CTS packet. *s*_1_ starts its transmission at time *D*, where *D* is a constant that is larger than the maximum propagation delay in the cluster. Based on equations (1) and (2), the minimal collision-free sending times of all source nodes can be calculated by:

(3) $$\left\{ \begin{array}{l} T_{1}^{data}=D\\ T_{j}^{data}=\max\{T_{i}^{data}+L_{i}^{data}+\max\{D_{s_{i}d_{j}}-D_{s_{j}d_{j}},D_{s_{i}d_{i}}-D_{s_{j}d_{i}}\}|i=1\sim j-1\}+C \end{array}\right..$$

where *C* is the guard time required to accommodate the variations of the propagation delays. Note that each node can obtain the same sending time table for all source nodes using Equation (3) if they receive all the CTS packets in the CTS stage correctly. Therefore, each source node can calculate its sending time independently without central control.

Similarly, all destination nodes reset their ACK stage clocks to the sending time of the last data packet which can be calculated using Equation (3). Then the sending time of the ACK/NACK packets can be calculated by:

(4) $$\left\{ \begin{array}{l} T_{1}^{ack}=D\\ T_{j}^{ack}=\max\{T_{i}^{ack}+L_{i}^{ack}+\max\{D_{d_{i}s_{j}}-D_{d_{j}s_{j}},D_{d_{i}s_{i}}-D_{d_{j}s_{i}}\}|i=1\sim j-1\}+C \end{array}\right.,$$

where $T_{1}^{ack}$ and $T_{i}^{ack}$ denote the sending time of the ACK/NACK packets from *d*_1_ and *d_i_*, respectively; $L_{i}^{ack}$ is the length of the ACK/NACK packet from *d_i_*; and $D_{d_{i}s_{i}}$, $D_{d_{i}s_{j}}$, $D_{d_{j}s_{j}}$ and $D_{d_{j}s_{i}}$ denote the delays from *d_i_* to *s_i_*, *d_i_* to *s_j_*, *d_j_* to *s_j_* and *d_j_* to *s_i_*, respectively.

#### 3.3.2. Sending Sequence Optimization

In Section 3.3.1, the optimized data sending time of each source node can be obtained using Equation (3) when the sending sequence is given. However, the total transmission time changes with the sending sequence due to the unequal propagation delays among different nodes. Therefore, the sending sequence should also be optimized to achieve better performance. If there are *N* pairs of nodes that succeed in handshaking in a transmission cycle, there will be *N*! sending sequence combinations, which requires a huge amount of computation when *N* becomes large. So, we propose a dynamic programming-based method to jointly optimize the sending sequence and time.

In the proposed method, the optimization problem is modeled by a state machine with *N* + 2 states. These states include the starting state, ending state, and state {*ST_i_*|1 ≤ *i* ≤ *N*} which correspond to the *N* source nodes. The state machine starts from the starting state and ends at the ending state. The starting state can transfer to state *ST_i_*. State *ST_i_* can transfer to the ending state. State *ST_i_* can transfer to state *ST_j_*, where i≠j, without any loops in the state transfer sequence. Define the cumulative weight of a state transfer sequence as the total transmission time. Then, the sending sequence optimization problem is equivalent to determine a state transfer sequence with the minimum cumulative weight. To simplify the discussion, we assume that all the data packets have the same sizes of *L^data^*. The proposed method can be implemented using the following steps:

1. Create *N* transition paths from the starting state to state *ST_i_*, 1 ≤ *i* ≤ *N*, and initialize the cumulative weights of these transition paths to 0. Set the iteration time *r* = 1.
2. For each surviving path, the states contained in this path are deleted from the state set $Q=\left\{ST_{1},ST_{2},\ldots ,ST_{N}\right\}$ to obtain a new state set *Q_k,r_*, where *k* is the index of the surviving path. Transfer the state machine from the current state to a new state in *Q_k,r_*. The sending time of the corresponding new node is given by:
  (5) $$T_{k,r}^{data}=\left\{ \begin{array}{l} D,\;r=1\\ \max\{T_{k,l}^{data}+L^{data}+\max\{D_{s_{kl}d_{kr}}-D_{s_{kr}d_{kr}},D_{s_{kl}d_{kl}}-D_{s_{kr}d_{kl}}\}|l=1\sim r-1\}+C,\;r>1 \end{array}\right.,$$
  where $T_{k,l}^{data}$ denotes the sending time of the *l*-th source node in the *k*-th surviving path. *s_kl_* and *d_kl_* denote the *l*-th source node and its corresponding destination node in the *k*-th surviving path, respectively; and $D_{s_{ki}d_{kj}}$ denote the propagation delay from *s_ki_* to *d_kj_*. The cumulative weight of the new path can be given by:
  (6) $$W_{k,r}^{data}=\max\{T_{k,i}^{data}+L^{data}+D+C|i=1\sim r\}.$$
3. Select the path with the least cumulative weight from all the paths that transfer to state *ST_i_*, 1 ≤ *i* ≤ *N*, as the surviving path and delete the others. Set the current state of each surviving path to the new state added in the *r*-th iteration.
4. If *r* < *N*, then r=r+1 and repeat steps 2–4 for another iteration. Otherwise, all surviving paths transfer from the current state to the ending state. The state sequence in the surviving path with the smallest cumulative weight is the optimized sending sequence.

The proposed method only has *N* iteration steps, so the calculation complexity is low. However, because the increased weight is not fixed as many dynamic programming problems and will change with the prior state sequence in a path, the proposed method does not guarantee obtaining the best solution.

### 3.4. Collisions

Although the calculation of the sending sequence and time of the source nodes can ensure that the data packets sent by a source node will not overlap with other packets at the corresponding destination node if the source nodes know the correct information about the source and destination node pairs in the current transmission cycle, collisions will still occur when a node loses one or more CTS packets. Assume that there are *M* destination nodes in the current transmission cycle and each destination node selects a time in $\left[0,T_{0}\right]$ to send its CTS packet uniformly and independently. Then for a source node *s_i_*, the arrival time of the CTS packets from the destination node *d_j_* will follow a uniform distribution in $\left[D_{d_{j}s_{i}},T_{0}+D_{d_{j}s_{i}}\right]$, i.e., $T_{d_{j}s_{i}}^{cts}\sim U\left(D_{d_{j}s_{i}},T_{0}+D_{d_{j}s_{i}}\right)$, where $T_{d_{j}s_{i}}^{cts}=T_{j}^{cts}+D_{d_{j}s_{i}}$ denotes the arrival time of the CTS packets from *d_j_* to *s_i_*; $T_{j}^{cts}$ and $D_{d_{j}s_{i}}$ are the sending time of the CTS packets from *d_j_* and the propagation delay from *d_j_* to *s_i_*, respectively; and U(*a*, *b*) denotes the uniform distribution. An approximate lower bound of the no-collision probability at *s_i_* is given in Appendix A as

(7) $$P\left(no\;collision\;at\;s_{i}\right)>1-M\left(M-1\right)\left(\frac{L^{cts}}{T_{0}}-\frac{1}{2}{\left(\frac{L^{cts}}{T_{0}}\right)}^{2}\right)$$

Equation (7) shows that the collision probability at *s_i_* is proportional to the second-order polynomial of *M* and become less when *L^cts^*/*T*_0_ decreases.

When one or more CTS packets are missing, a source node will miscalculate its sending time table, which will cause collisions in the current transmission cycle. Let $T_{i-err}^{data}$ denote the miscalculated sending time of *s_i_*. Then, the data packet from *s_i_* will conflict with the packet from *s_j_* when:

(8) $$T_{i\_err}^{data}>T_{j}^{data}+D_{s_{j}d_{i}}-D_{s_{i}d_{i}}-L^{data}$$

or:

(9) $$T_{i\_err}^{data}<T_{j}^{data}+L^{data}+D_{s_{J}d_{J}}-D_{s_{i}d_{J}}.$$

This miscalculation only affects the current transmission cycle and will not accumulate.

### 3.5. Multi-Hop Extension

Although the proposed method is designed for one-hop clusters, it can also be applied to multi-hop networks. When two clusters overlap each other, the nodes belong to both clusters can only joint the communication of one cluster, which will affect the communication of the other if they send out packets. So the simplest application of the proposed method to the multi-hop network is to stop the transmission of a cluster if the communication of any other overlapping clusters will affect it. This scheme can be summarized as:

1) When the nodes that belong to multiple clusters are idle, all clusters can communicate independently.
2) If a node that belongs to multiple clusters has data to send to a destination node in one cluster, it must wait for the nodes in other clusters to be idle and then joins a transmission cycle in the destination cluster. The nodes in the other clusters stop trying to communicate when they receive the RTS packet from the overlapping node and wait until it finishes sending the data packet, or timeout.
3) If a node that belongs to multiple clusters receives an RTS packet from one cluster, it needs to check the state of the other clusters. The CTS packet can only be sent out when all the other clusters are idle. The nodes in the other clusters stop trying to communicate when they receive this CTS packet and wait until they receive the ACK/NACK packet sent by the overlapping node.

This scheme does not need to synchronize the overlapping clusters, which is difficult to implement in underwater environments. However, because the overlapping clusters cannot work concurrently when there are nodes belonging to multiple clusters needing to join the communication of one cluster, the transmission efficiency in the multi-hop situation is not as high as in the one-hop situation. More sophisticated synchronization or using multi-channel may improve the performance of the proposed method in multi-hop environments. These technologies are beyond the scope of this paper.

## 4. Simulations and Results

The simulations were implemented by Aqua-Sim [22], which is an NS-2 based simulator for underwater sensor networks and developed at the Underwater Sensor Network (UWSN) Lab at the University of Connecticut. In the experiment, the nodes are randomly located in a square area 1.5 × 1.5 km^2^ and 500m deep. All nodes in the cluster are static and can reach the other nodes directly. In the network initialization stage, each node measures the round-trip time with other nodes, and broadcasts this information to the others to obtain an inter-node propagation delay table. The acoustic propagation speed is set to 1500 m/s. Data packet size, control packet size, and data rate are 125 bytes, 10 bytes and 1200 bps, respectively. The powers of sending, receiving, and idle states are set to 20, 1 and 0.5 Watt, respectively. Data packets generation for each node follows a Poisson distribution with parameter λ packets/s, and the destination of each packet is selected randomly with equal probability. Channel-related packet losses are ignored, i.e., packet losses only occur due to packet collisions. In the simulations, the normalized throughput, packet delivery rate, average end-to-end delay, and average energy consumption of the proposed method are compared with underwater ALOHA (UWALOHA), underwater carrier sense multiple access/collision avoidance (UW-CSMA/CA), slotted floor acquisition multiple access (SFAMA) and ROPA protocols with different network loads and node numbers. The normalized throughput, packet delivery rate, average end-to-end delay, average energy consumption and normalized offered load are defined as [23]:

- Normalized throughput: The ratio of the average number of data bits successfully received by the intended destinations per second to the transmission bit rate (dimensionless).
- Packet delivery rate: The ratio of the number of data packets successfully delivered at the intended destinations to the total number of data packets generated (dimensionless).
- Average end-to-end delay: The average time interval between generation and successful delivery of data packets at the intended destinations (in ms).
- Average energy consumption: The ratio of the total energy consumption to the number of data packets successfully received by the intended destinations (in Joule/packet or J/pkt).
- Normalized offered load: The ratio of the average number of data bits sent per second to the transmission bit rate (dimensionless).

UWALOHA, UW-CSMA/CA, slot SFAMA are typical ALOHA, CSMA, and handshaking based MAC protocols respectively. ROPA is an underwater MAC protocol that employs the temporal reuse technique to support multiple data transmissions in a transmission cycle.

### 4.1. Performance Under Different Network Loads

**Figure 3:**
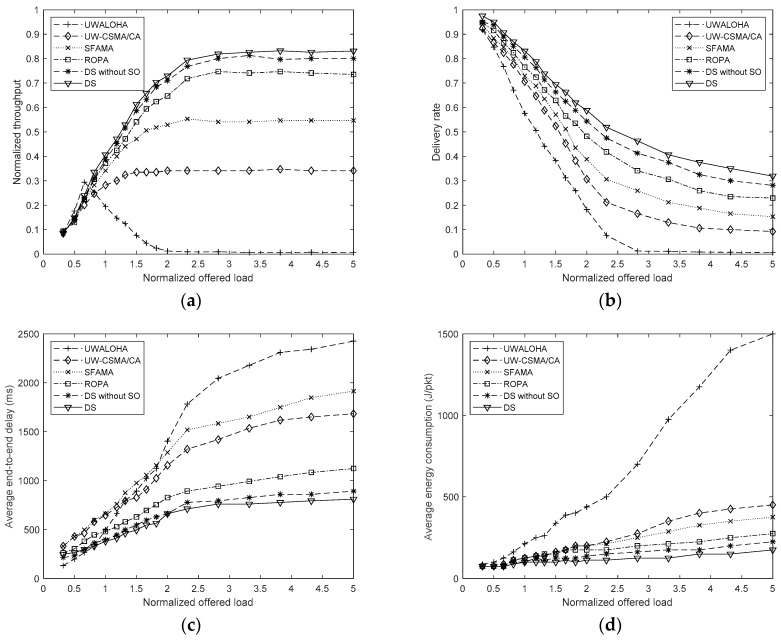
Performance of UWALOHA, UW-CSMA/CA, SFAMA, and ROPA protocols and that of the proposed method under different network loads. (**a**) Normalized throughput; (**b**) Packet delivery rate; (**c**) Average end-to-end delay; (**d**) Average energy consumption.

The performance of UWALOHA, UW-CSMA/CA, SFAMA, and ROPA protocols, and the proposed method under different network loads are shown in Figure 3, where “DS” and “DS without SO” denote the proposed distributed scheduling method with and without sending sequence optimization, respectively.

Figure 3a shows the normalized throughputs of these protocols under different network loads. Figure 3a shows that the proposed method reaches its maximum normalized throughput around 0.85 when the network load is higher than 2.8. The normalized throughputs of DS and DS without SO are both higher than the four conventional MAC protocols. DS produces higher normalized throughput than DS without SO, which shows the effectiveness of the proposed joint optimization method. UWALOHA provides the highest normalized throughput when network load is 0.3–0.6, but decreases rapidly as the network load increases due to the massive conflicts caused by uncontrolled transmission. UW-CSMA/CA and SFAMA reach their maximum normalized throughputs around 0.35 and 0.56 under the network loads of 1.5 and 2.3, respectively. The maximum normalized throughputs of UW-CSMA/CA and SFAMA are much lower than those of the proposed method because, in UW-CSMA/CA and SFAMA, only one pair of nodes can communicate in a transmission cycle. This means that a node needs to compete for the transmission opportunity using the RTS/CTS handshake mechanism for each data packet, which significantly reduces the channel use in underwater environments. When only one node needs to transmit data, the proposed method provides the same performance as UW-CSMA/CA and SFAMA. However, when there is more than one node that has data to send, the proposed method can shorten the data transmission time by optimizing the transmission schedules of each source node, and the concurrent handshaking also reduces the overhead. Therefore, the proposed method can support heavier network loads and achieve higher maximum normalized throughput than UW-CSMA/CA and SFAMA. ROPA has similar normalized throughputs as the proposed method when the network load is around 0.3–1.3 but becomes lower as the network load increases because in ROPA, additional concurrent transmissions only occur when other nodes have data to send to the source node, which will sometimes reduce the concurrent transmission opportunity. The proposed method imposes no constraints on the source and destination nodes and can support multiple transmissions with optimized sequences and time in a transmission cycle, so that can provide better performance than ROPA.

Figure 3b shows the delivery rates of the four conventional protocols and that of the proposed method. The delivery rates of all methods drop as the network load increases. UWALOHA provides the lowest delivery rate and tends to be zero when the network load is higher than 2.8. The delivery rates of UW-CSMA/CA, SFAMA, and ROPA are higher than UWALOHA because they use handshaking to avoid conflicts. The proposed method provides the highest delivery rate in the simulation because collisions only occur when a node loses the CTS packets and the collision rate of the CTS packets is usually low due to their small size. Concurrent transmission can also improve the total number of transmitted data packets in a fixed time interval, which produces a higher delivery rate. DS had a higher delivery rate than DS without SO because the former can transmit more data packets than the latter in the same time interval under the same collision rates of the CTS packets.

Figure 3c shows the average end-to-end delays of the four conventional protocols and that of the proposed method. UWALOHA produced the lowest average delay when the network load was below 0.6 but worsened in high network load environments due to the collisions and retransmission. UW-CSMA/CA and SFAMA produced high average delays because handshaking is needed for each data packet transmission. The average delay of ROPA was significantly smaller than those of UW-CSMA/CA and SFAMA because it supports concurrent transmission of multiple nodes in a transmission cycle so that the average handshaking overhead is reduced. However, the nodes whose destinations are not the source node still need to wait for another transmission cycle, which limits the performance of ROPA. Compared with ROPA, the proposed method increases the opportunity of concurrent transmission in a transmission cycle, so had the lowest average delay in the simulation. DS results in lower average delay than DS without SO due to the more compatible transmission scheduling.

Figure 3d shows the average energy consumptions of the four conventional protocols and that of the proposed method. UWALOHA consumes the highest average energy due to the retransmissions caused by the frequent collisions. UW-CSMA/CA and SFAMA have medium average energy consumption because handshaking reduces the collision while introducing the additional transmission of RTS and CTS packet for each data packet. ROPA and the proposed method reduce the average overhead energy consumption by supporting concurrent transmission in each transmission cycle. The increase in the concurrent transmission opportunity in the proposed method leads to lower average overhead energy consumption than ROPA. DS results in lower average energy consumption than DS without SO.

### 4.2. Performance with Different Network Sizes

**Figure 4:**
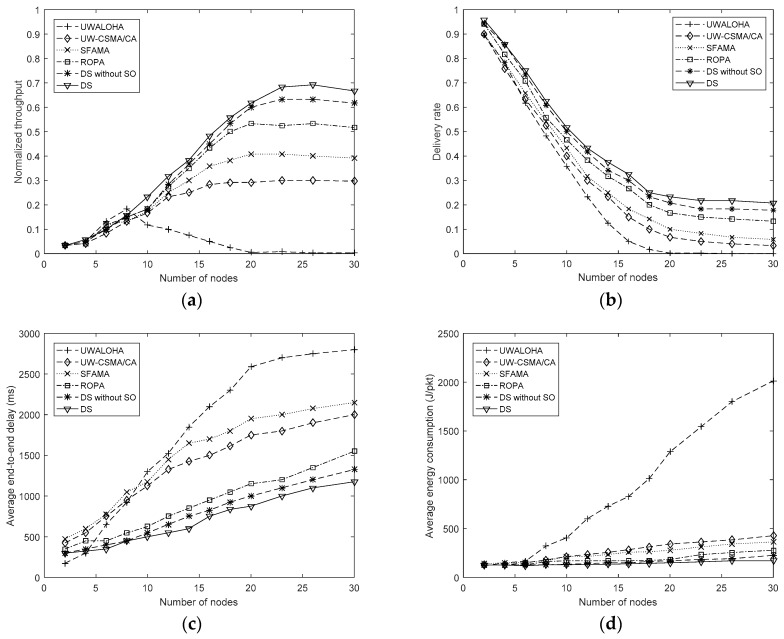
Performance of UWALOHA, UW-CSMA/CA, SFAMA, and ROPA protocols and that of the proposed method under different network sizes. (**a**) Normalized throughput; (**b**) Packet delivery rate; (**c**) Average end-to-end delay; (**d**) Average energy consumption.

The performance of UWALOHA, UW-CSMA/CA, SFAMA, and ROPA, and that of the proposed method under different network sizes are shown in Figure 4. The arrival rates of the Poisson distribution were set to 100 in all simulations.

Figure 4a shows that UWALOHA achieves the highest throughput when the number of nodes is less than 8 but drops quickly as the network size increases. UW-CSMA/CA, SFAMA, and ROPA reach their maximum throughput of 0.30, 0.40, and 0.53, respectively, at the network size of 20. The throughputs of DS and DS without SO become saturated after the network size reaches 26. They achieved the maximum throughputs of 0.70 and 0.65, respectively, which were the highest in the simulation, because the proposed method provides more concurrent transmission opportunity so that more nodes can transmit their data in one transmission cycle. DS also outperformed DS without SO in the simulation results.

In Figure 4b, the delivery rates of all methods decrease as the network size increases because the network load increases with the network size. UWALOHA had the lowest delivery rate and tended to be zero when the network size was larger than 20. The delivery rates of UW-CSMA/CA, SFAMA and ROPA were higher than that of UWALOHA. DS results in the highest delivery rate in the simulation.

Figure 4c shows the average end-to-end delays of the four conventional protocols and that of the proposed method. The average delays of all methods drop as the network size increases because larger networks introduce more collisions. UWALOHA works better than the others when the network size is below four and better than UW-CSMA/CA and SFAMA when the network size is below eight. This occurs because no overhead is needed in UWALOHA. However, when the network size becomes large, its average delay increases significantly due to more collisions and retransmissions. UW-CSMA/CA and SFAMA are worse than ROPA and the proposed method because of their serial transmission modes. The proposed method outperforms ROPA in different network sizes.

Figure 4d shows the average energy consumption of the four conventional protocols and that of the proposed method. UWALOHA has the highest average energy consumption, especially when the network size becomes large. The handshaking can effectively reduce collision so that average energy consumptions of the handshaking based protocols are much smaller than UWALOHA in large-scale networks. Among the four handshaking-based protocols, DS resulted in the lowest energy consumption in almost all the network sizes.

## 5. Discussion

The simulation results show that UWALOHA works well with low network loads and small network sizes but performance worsens quickly as the network load or size increase. This occurs because without control packets, UWALOHA can reduce the transmission overhead but produces more collisions and retransmissions when the packet traffic increases. In UW-CSMA/CA and SFAMA, handshaking effectively avoids collisions, so these protocols outperform UWALOHA under high network loads and large network sizes. However, the RTS/CTS control packets introduce transmission overhead, which reduces the performance under low network load and small network size. Handshaking also increases the delay in transmission. ROPA allows additional concurrent transmission to the source node after the source node transmits its data, so its performance is better than UW-CSMA/CA and SFAMA under different network loads and network sizes. Compared with ROPA, the proposed method supports arbitrary multi-pair concurrent transmission, which increases transmission opportunity in a transmission cycle. Therefore, it produced the best performance in normalized throughput, delivery rate, average end-to-end delay, and average energy consumption under different network loads and network sizes in the simulations. The simulation results also show that the proposed method, with sending sequence optimization, outperforms that without sending sequence optimization.

Although the proposed method works well in the one-hop cluster, several aspects of its performance can be further improved. First, in Equations (1) and (2), we assumed that if *s_i_* sends out its data packet before *s_j_*, then the data packets from *s_i_* will arrive at *d_i_* and *d_j_* earlier than the data packets from *s_j_*. This assumption sets a constraint on the packet arrival sequence at the destination node, which simplifies the question but leads to a sub-optimal solution. Further improvement can be expected by removing this constraint. Second, in the proposed method, collisions of data packets still occur if a node loses one or more CTS packets. Any method that can reduce or eliminate the collision of CTS packets will improve the performance of the proposed method. Third, the proposed method is developed for one-hop clusters. Although it can be extended to multi-hop situations, the performance will worsen when nodes belonging to multiple clusters join the communication. Therefore, inter-cluster optimization should be considered for multi-hop networks.

## 6. Conclusions

In this paper we propose a new distributed scheduling method for underwater acoustic networks that supports multiple handshakes and concurrent transmissions in one transmission cycle for one-hop clusters. The proposed method is based on the two-way handshake mechanism; a deterministic scheduling algorithm was developed to calculate the sending time table for each source node. The proposed deterministic scheduling algorithm optimizes the sending sequence and time of the source nodes jointly by dynamic programming to ensure packets sent by a source node do not conflict with other packets at the corresponding destination node, while the whole data transmission time is as short as possible. Using the deterministic scheduling algorithm can also effectively reduce the scheduling overhead. Simulation results showed that the proposed method outperforms UWALOHA, UW-CSMA/CA, slot SFAMA, and ROPA protocols in normalized throughput, packet delivery rate, average end to end delay, and average energy consumption under different network loads and sizes.

## Figures and Tables

**Figure A1:**
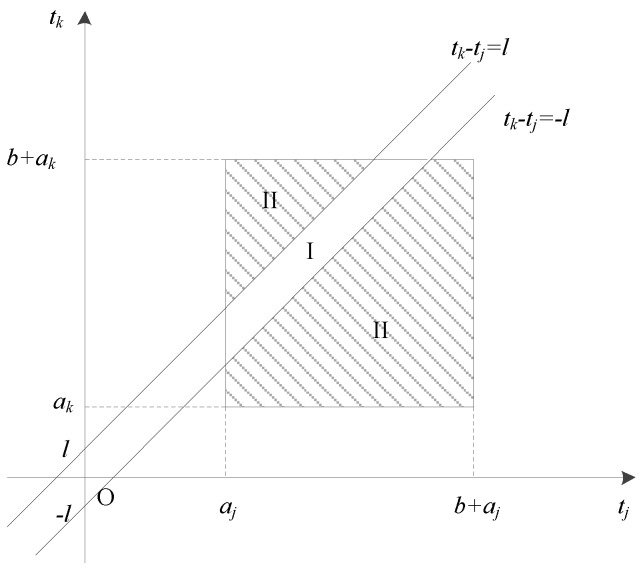
A section of the collision and collision-free parts on the $t_{j}-o-t_{k}$ plane.

## Appendix A

To simplify the expression, let *t_j_* denotes $T_{d_{j}s_{i}}^{cts}$, *a_j_* denotes $D_{d_{j}s_{i}}$, *b* denotes *T*_0_, *l* denotes *L^cts^*, where 1 ≤ *j* ≤ *M*. Then *t*_1_, *t*_2_, …, *t_M_* are *M* independent random variables that follow the uniform distributions of $U\left(a_{j},b+a_{j}\right)$. Therefore the point $\left(t_{1},t_{2},\ldots ,t_{M}\right)$ is located in a hypercube with the side length of *b* in *M*-dimensional space and has a probability density function of $p\left(t_{1},t_{2},\ldots ,t_{M}\right)=\frac{1}{b^{M}}$. Collisions will occur when $\left|t_{j}-t_{k}\right|<l$, where 1 ≤ *j* ≤ *M*, 1 ≤ *k* ≤ *M* and *j* ≠ *k*. Then, the hypercube is divided into two parts by the hyper-planes of $t_{k}-t_{j}=l$ and $t_{k}-t_{j}=-l$. Part I is the union of the regions in the hypercube that are sandwiched between $t_{k}-t_{j}=l$ and $t_{k}-t_{j}=-l$. When $\left(t_{1},t_{2},\ldots ,t_{M}\right)$ falls into this part, a collision will occur. Part II is the union of the regions in the hypercube that are outside $t_{k}-t_{j}=l$ and $t_{k}-t_{j}=-l$. $\left(t_{1},t_{2},\ldots ,t_{M}\right)$ belongs to this part, meaning it is collision-free. Figure A1 shows a section of these two parts on the $t_{j}-o-t_{k}$ plane. The sectional area of part II in the $t_{j}-o-t_{k}$ plane will reach its minimum when the diagonal of the cube overlaps with line $t_{k}-t_{j}=0$, i.e., $a_{j}=a_{k}$.

Let $\Phi^{I}$ and $\Phi^{\mathrm{II}}$ denote parts I and II of the hypercube, respectively. Then, the probability of no collision can be given by:

(A1) $$P\left(no\;collision\right)=\underset{\Phi^{\mathrm{II}}}{\int \ldots }\int \frac{1}{b^{M}}dt_{1}dt_{2}\ldots dt_{M}=1-\underset{\Phi^{I}}{\int \ldots }\int \frac{1}{b^{M}}dt_{1}dt_{2}\ldots dt_{M}$$

Because $\Phi^{I}$ and $\Phi^{\mathrm{II}}$ are piecewise functions of *a_j_*, it is hard to obtain a closed expression for this probability. However, the probability of no collision will reach a minimum when *a_j_* = *A*, where 1 ≤ *j* ≤ *M* and A is a constant. If we ignore the overlapping parts among the sandwiched regions of $t_{k}-t_{j}=l$ and $t_{k}-t_{j}=-l$ in the hypercube, where 1 ≤ *j* ≤ *M*, 1 ≤ *k* ≤ *M* and *j* ≠ *k*, there will be

(A2) $$\begin{array}{cc} P\left(no\;collision\right) & >1-\sum_{1\leq j,k\leq M,j\neq k}\left(\iint_{\Phi_{jk}^{I}}\frac{1}{b^{2}}dt_{j}dt_{k}\underset{1\leq l\leq M,l\neq j,l\neq k}{\underset{︸}{\cdot \int_{A}^{A+b}\frac{1}{b}dt_{1}\cdots \int_{A}^{A+b}\frac{1}{b}dt_{l}\cdots \int_{A}^{A+b}\frac{1}{b}dt_{M}}}\right)\\ & =1-\sum_{1\leq j,k\leq M,j\neq k}\frac{2bl-l^{2}}{b^{2}}\\ & =1-C_{M}^{2}\left(\frac{2l}{b}-\frac{l^{2}}{b^{2}}\right)\\ & =1-\frac{M!}{\left(M-2\right)!2!}\cdot \left(\frac{2l}{b}-\frac{l^{2}}{b^{2}}\right)\\ & =1-M\left(M-1\right)\left(\frac{l}{b}-\frac{l^{2}}{2b^{2}}\right) \end{array}$$

where $\Phi_{jk}^{I}$ denote the sectional region of part I in the $t_{j}-o-t_{k}$ plane. Equation (A2) gives an approximate lower bound for the no-collision probability at $s_{i}$.
